# Associations between risk perception, spontaneous adaptation behavior to heat waves and heatstroke in Guangdong province, China

**DOI:** 10.1186/1471-2458-13-913

**Published:** 2013-10-02

**Authors:** Tao Liu, Yan Jun Xu, Yong Hui Zhang, Qing Hua Yan, Xiu Ling Song, Hui Yan Xie, Yuan Luo, Shannon Rutherford, Cordia Chu, Hua Liang Lin, Wen Jun Ma

**Affiliations:** 1 Guangdong Provincial Institute of Public Health, Panyu District, Guangzhou 511430, China; 2 Guangdong Provincial Center for Disease Control and Prevention, Panyu District, Guangzhou 511430, China; 3 Environment and Health, Guangdong Provincial Key Medical Discipline of Twelfth Five-Year Plan, Panyu District, Guangzhou 511430, China; 4 Center for Environment and Population Health, Griffith University, Brisbane, QLD 4111, Australia

**Keywords:** Risk perception, Climate change, Heat waves, Extreme heat, Adaptation behaviors, Heatstroke, Interactive effect

## Abstract

**Background:**

In many parts of the world, including in China, extreme heat events or heat waves are likely to increase in intensity, frequency, and duration in light of climate change in the next decades. Risk perception and adaptation behaviors are two important components in reducing the health impacts of heat waves, but little is known about their relationships in China. This study aimed to examine the associations between risk perception to heat waves, adaptation behaviors, and heatstroke among the public in Guangdong province, China.

**Methods:**

A total of 2,183 adult participants were selected using a four-stage sampling method in Guangdong province. From September to November of 2010 each subject was interviewed at home by a well-trained investigator using a structured questionnaire. The information collected included socio-demographic characteristics, risk perception and spontaneous adaptation behaviors during heat wave periods, and heatstroke experience in the last year. Chi-square tests and unconditional logistic regression models were employed to analyze the data.

**Results:**

This study found that 14.8%, 65.3% and 19.9% of participants perceived heat waves as a low, moderate or high health risk, respectively. About 99.1% participants employed at least one spontaneous adaptation behavior, and 26.2%, 51.2% and 22.6% respondents employed <4, 4–7, and >7 adaptation behaviors during heat waves, respectively. Individuals with moderate (OR=2.93, 95% CI: 1.38-6.22) or high (OR=10.58, 95% CI: 4.74-23.63) risk perception experienced more heatstroke in the past year than others. Drinking more water and wearing light clothes in urban areas, while decreasing activity as well as wearing light clothes in rural areas were negatively associated with heatstroke. Individuals with high risk perception and employing <4 adaptation behaviors during heat waves had the highest risks of heatstroke (OR=47.46, 95% CI: 12.82-175.73).

**Conclusions:**

There is a large room for improving health risk perception and adaptation capacity to heat waves among the public of Guangdong province. People with higher risk perception and fewer adaptation behaviors during heat waves may be more vulnerable to heat waves.

## Background

The fourth report of the Intergovernmental Panel on Climate Change (IPCC) stated that heat waves have become more frequent in the past half century, and projected that the world’s surface temperature will continue to rise with more frequent extreme heat events in the next several decades [1]. Many studies throughout the world have reported a significant association between elevated temperature and mortality [2,3]. For example, the unprecedented European heat wave in the summer of 2003 was estimated to have led to 22,000-35,000 premature deaths [4], and the 2003 heat wave in Shanghai was also found to be linked with higher total, cardiovascular and respiratory mortality risks [5]. Risk perception and adaptation are two important components in reducing the health impacts of climate change [6,7]. Therefore, there is a need to better understand the public’s perception of the health risks of heat waves and their adaptation behaviors to these events when making adaptation policies targeted at vulnerable populations.

Health risk perception can be defined as the subjective assessment of the probability of a specified type of accident happening and how concerned we are with the consequences [6]. For this study, adaptation behavior refers to adjustments to moderate potential damage, to take advantage of opportunities or to cope with the consequences of heat waves [7]. A direct effect of adaptation behavior can reduce vulnerability. Therefore, a climate hazard may have little or no health impact if it occurs in a population that can adapt to or cope adequately with it.

Research on disaster management suggests that risk perception and adaptation actions are closely related [8-10]. In the context of heat waves, Kalkstein et al. found that elevated risk perception to heat waves among the public resulted in increased changes in daily activity to reduce the health impacts of heat, such as heatstroke [11,12]. Heatstroke is a life-threatening illness characterized by elevation of core body temperature above 40°C and central nervous system deregulation culminating in coma [13,14]. Heatstroke may progress to multiple organ failure, such as rhabdomyolysis, acute renal failure, acute hepatic failure, elevation of serum pancreatic enzymes, myocardial injury and disseminated intravascular coagulopathy, and even death [13,14].

To reduce heatstroke incidence and heat wave related mortality, it is essential to better understand the risk perception to heat waves, adaptation behaviors during extreme heat events, and their associations with heatstroke incidence. However, few studies have been conducted in China [15-17], though results from such a study would be important in developing strategies to reduce the health impacts of heat waves.

A population-based cross-sectional survey was conducted in Guangdong province, China in 2010 to identify the risk perception to heat waves, identify the type and extent of individual adaptation behaviors to heat waves, and analyze the relationships between perception, adaptation behavior and heatstroke. This research is an important starting point for developing evidence-based risk communication strategies relating to heat waves, including identifying target populations, raising awareness and suggesting effective adaptation behaviors.

## Method

### Subjects

**Figure 1:**
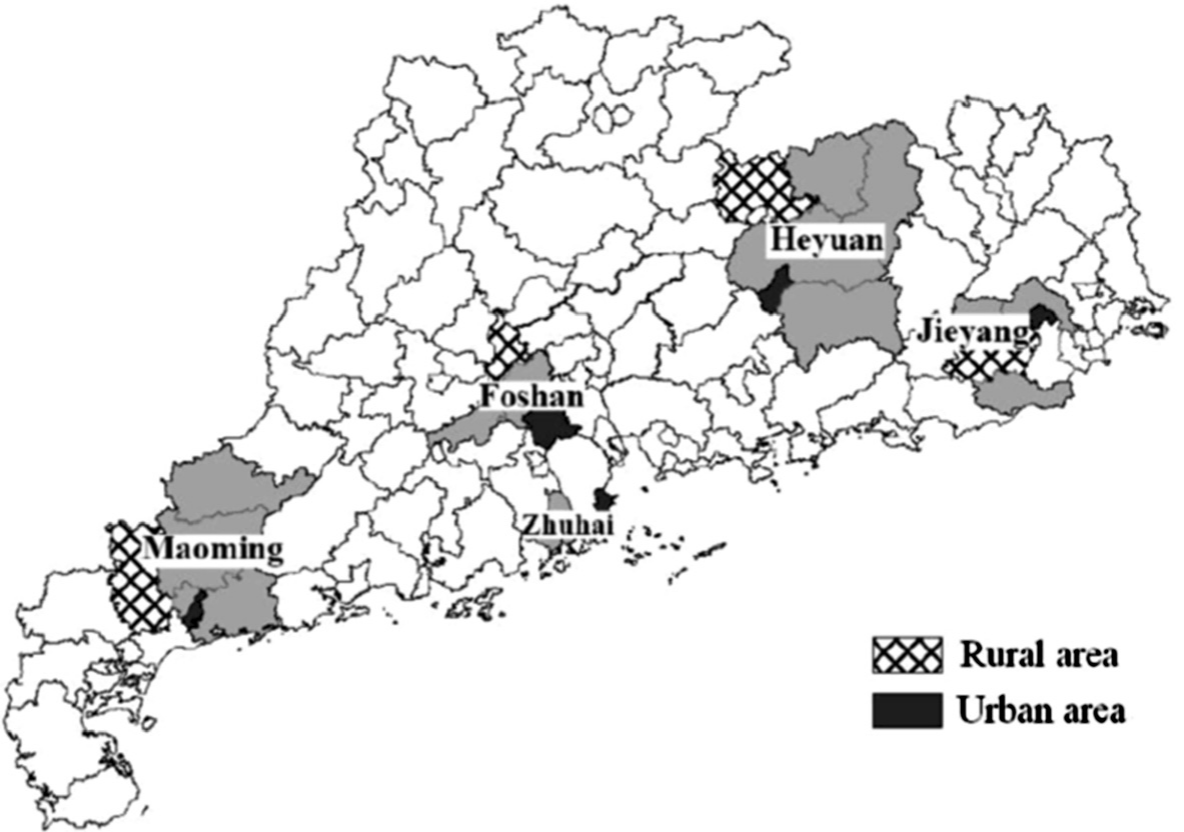
**Mapping the sample selection in Guangdong Province, China.***Note*: In each selected city, one county and one district were sampled to represent the rural and urban area. The five counties selected in Zhuhai, Foshan, Maoming, Heyuan and Jieyang were Jianwan, Sanshui, Huazhou, Lianping and Puning, respectively. Correspondingly, the five districts selected in the five cities were Xiangzhou, Shunde, Maogang, Yuancheng and Rongcheng, respectively.

A four-stage sampling method was employed to select a representative sample in Guangdong province, China. First, the province was geographically divided into east, south, west, north and central areas, with one city randomly selected in each area. The five selected cities were Zhuhai, Foshan, Maoming, Heyuan and Jieyang (Figure 1). In the second stage, one county and one district were randomly selected in each selected city to represent rural and urban locations, respectively. In the third stage, all villages and communities in each selected county and district were further divided into sub-areas, each of which had about 50 households. Six sub-areas were randomly selected in each county and district. Finally, in each selected sub-area, all residents aged over 15 years who had lived in that community for at least 6 months were registered, and one eligible local resident in each household was selected using the KISH grid method [18].

### Data collection

Trained interviewers used a standardized structured questionnaire to interview all the sampled subjects at home between September and November of 2010. Before the interview, all interviewers received intensive, systematic training. Firstly, a pilot study was conducted in Heshan (a city in Guangdong province) to ensure the interviewers clearly understood the questionnaire and the survey procedure, and to test the reliability and validity of the survey questionnaire. Then, the research team discussed modifications to the research procedure and questionnaire. The information collected through the questionnaires included socio-demographic characteristics (age, gender, occupation, education, household income, and living location), health risk perception to heat waves, spontaneous adaptation behaviors to heat waves in the past year and whether the individual had ever experienced heatstroke in the past one year (see Additional file 1).

Approval to conduct this study was granted by the ethics committee of the Guangdong Provincial Center for Disease Control and Prevention in Guangzhou, China, and a written informed consent was obtained from each participant before the interview.

### Measurement of variables

According to the Chinese Meteorological Administration’s definition, a “heat day” is defined as a day with maximum temperature exceeding 35°C, and a heat wave is a period of at least three consecutive “heat days” [5].

Health risk perception to heat waves was assessed by one question: How dangerous are heat waves to your health? The response options were on a scale from 1 (absolutely none) to 7 (extreme), such that a higher number was indicative of a greater risk perception [11,19]. Participants were divided into three groups according to their reported perception: low risk to health (response of 1 or 2), moderate risk to health (response of 3, 4 or 5), and high risk to health (response of 6 or 7).

Each interviewed participant was asked to identify their commonly used spontaneous adaptation behaviors during the heat waves. Participants were asked to choose from a list of adaptation behaviors that included opening windows, drinking more water, wearing light clothes, bathing more frequently, staying in house, reducing activity, going to a public place with air conditioning, resting in the shade, using an air conditioner, using a sunshade or sunhat, and none [11].

To identify the occurrence of heatstroke, every participant was asked whether they had been diagnosed with heatstroke by a clinical doctor or had any symptoms of heatstroke during heat waves in the past one year. Symptoms included dizziness, headache, nausea, vomiting, chest stuffiness, palpitation, and muscle spasms. If they were diagnosed by a doctor, or reported experiencing any of the symptoms and could exclude other reasons for such symptoms, such as an intestinal infection, they were defined as having experienced a heatstroke.

### Statistical analysis

Means and standard deviations (SD) were calculated for continuous variables such as age. Categorical variables were calculated as a percentage of subjects with the respective attribute. Firstly, we used a series of Chi-square tests to examine the associations of heatstroke experience in the past one year (0=no, 1=yes), health risk perception (0=low impact, 1=moderate impact, and 2=high impact) and spontaneous adaptation behaviors to heat waves with socio-demographic characteristics. Secondly, we employed several unconditional logistic regression models to examine the associations of each spontaneous adaptation behavior with heatstroke, and subgroup analyses were also conducted in urban and rural areas. Thirdly, in order to test the interactive effects between perception and spontaneous adaptation behaviors to heatstroke during heat waves, we counted every participant’s number of adaptation behaviors, and divided them into three groups according to their distributions (≤3, 4–7, and ≥8 adaptation behaviors). Then, several unconditional logistic regression models were used to assess the associations of health risk perception and number of adaptation behaviors to heat waves with heatstroke after adjustment for potential confounders. A product term of health risk perception and number of adaptation behaviors was further added into the logistic model to test their joint effect on heatstroke. The adjusted confounders included age, gender, education, occupation, household income and residential area.

All statistical analyses were two-sided, and values of P<0.05 were considered statistically significant. All analyses were conducted with IBM SPSS Statistics 19.0 software.

## Results

### Socio-demographic characteristics of the participants

A total of 2,183 adults were included in the present study, in which 53.4% of the participants were males, and 51.3% of the participants were from rural areas. About 60% of respondents had an education level below junior middle school, and about half of the families had a per capita monthly income of less than 2000 yuan. The average age of the participants was 39.3 (SD=14.2) years. Other detailed socioeconomic characteristics were depicted in Table 1.

**Table 1:** Demographic characteristics of 2183 participants from Guangdong province

	**Heatstroke experience**		***χ***^**2**^	**P**
	**Yes (n=2,034)**	**No (n=149)**		
	**N (%)**	**N (%)**		
**Gender**			0.06	0.805
Male	1,087 (93.3)	78 (6.7)		
Female	947 (93.0)	71 (7.0)		
**Age (years)**			3.81	0.580
<20	168 (94.34)	10 (5.6)		
20–29	401 (93.3)	29 (6.7)		
30–39	495 (93.6)	34 (6.4)		
40–49	479 (93.6)	33 (6.5)		
50–59	282 (90.7)	29 (9.3)		
≥60	209 (93.7)	14 (6.3)		
**Education**			7.18	0.127
Elementary school or lower	474 (91.2)	46 (8.9)		
Junior middle school	652 (92.6)	52 (7.4)		
Senior middle school or vocational school	493 (94.8)	27 (5.2)		
College or above	355 (94.7)	20 (5.3)		
Didn’t answer	60 (93.8)	4 (6.3)		
**Occupation**			30.13	<0.001
Agriculture, forestry, animal husbandry or fishing	449 (88.6)	58 (11.4)		
Service trade	417 (93.1)	31 (6.2)		
Person in charge of institute	145 (95.4)	7 (4.6)		
Technician	243 (96.4)	9 (3.6)		
Military or student	166 (93.3)	12 (6.7)		
Unemployment or retired	236 (92.6)	19 (7.5)		
Other	378 (96.7)	13 (3.3)		
**Family monthly income per person (yuan)**			39.54	0.001
< 500	246 (85.4)	42 (14.6)		
500–999	350 (91.4)	33 (8.6)		
1,000–1,999	396 (96.1)	16 (3.9)		
2,000–4,999	491 (94.6)	28 (5.4)		
5,000–9,999	159 (94.1)	10 (5.9)		
≥10,000	64 (97.0)	2 (3.0)		
Don’t know or refused to answer	328 (94.8)	18 (5.2)		
**Residential area**			38.60	<0.001
Urban	1,028 (96.6)	36 (3.4)		
Rural	1,006 (89.9)	113 (10.1)		

The percentages of heat stroke experienced in the past one year were significantly higher in people employed in agriculture, forestry, animal husbandry or fishing (11.4%), compared with other occupations. Those with lower family incomes (14.6% in <500 yuan of per person) and from rural areas (10.10%) had a higher heatstroke incidence than those with higher incomes or from urban areas.

### Risk perception and spontaneous adaptation behaviors to heat waves

In the sampled population 14.8%, 65.3% and 19.9% of individuals reported that heat waves presented a low, moderate or high threat to their health, respectively. Table 2 shows that risk perception to heat waves was significantly lower in males, young people (<30 years) and the elderly (50~59 years), people with less education (below junior middle school), people who had a extremely low income (<500 yuan per person) or an extremely high family income (≥10,000 yuan per person), and those employed in agriculture, forestry, animal husbandry or fishing, and from rural areas.

**Table 2:** Self-reported risk perception to heat waves in 2,183 participants from Guangdong province

**Variables**	**Health risk perception of heat waves**			***χ***^**2**^	**P**
	**Low impact**	**Moderate impact**	**High impact**		
	**N (%)**	**N (%)**	**N (%)**		
**Gender**				8.62	0.013
Male	194(16.7)	731(62.7)	240(20.6)		
Female	130(12.8)	694(68.2)	194(19.1)		
**Age (years)**				33.02	<0.001
<20	40(22.5)	114(64.0)	24(13.5)		
20–29	69(16.0)	293(68.1)	68(15.8)		
30–39	62(11.7)	335(63.3)	132(25.0)		
40–49	82(16.0)	317(61.9)	113(22.1)		
50–59	43(13.8)	218(70.1)	50(16.1)		
≥60	28(12.6)	148(66.4)	47(21.1)		
**Education**				108.97	<0.001
Elementary school or lower	67(12.9)	374(71.9)	79(15.2)		
Junior middle school	111(15.8)	492(69.9)	101(14.3)		
Senior middle school or vocational secondary school	95(18.3)	323(62.1)	102(19.6)		
College or above	41(10.9)	191(50.9)	143(38.1)		
Didn’t answer	10(15.6)	45(70.3)	9(14.1)		
**Occupation**				75.72	<0.001
Agriculture, forestry, animal husbandry or fishing	60(11.8)	375(74.0)	72(14.2)		
Service trade	61(13.6)	312(69.6)	75(16.7)		
Person in charge of institute	28(18.4)	77(50.7)	47(30.9)		
Technician	37(14.7)	130(51.6)	85(33.7)		
Military or student	34(19.1)	114(64.0)	30(16.9)		
Unemployment or retired	33(12.9)	173(67.8)	49(19.2)		
Other	71(18.2)	244(62.4)	76(19.4)		
**Family monthly income per person (yuan)**				57.00	<0.001
< 500	35(12.2)	205(71.2)	48(16.7)		
500–999	68(17.8)	245(64.0)	70(18.3)		
1,000–1,999	53(12.9)	253(61.4)	106(25.7)		
2,000–4,999	71(13.7)	310(59.7)	138(26.6)		
5,000–9,999	32(18.9)	108(63.9)	29(17.2)		
≥10,000	11(16.7)	49(74.2)	6(9.1)		
Don’t know or refused to answer	54(15.6)	255(73.7)	37(10.7)		
**Residential area**				134.49	<0.001
Urban	159(14.9)	588(55.3)	317(29.8)		
Rural	165(14.7)	837(74.8)	117(10.5)		

**Figure 2:**
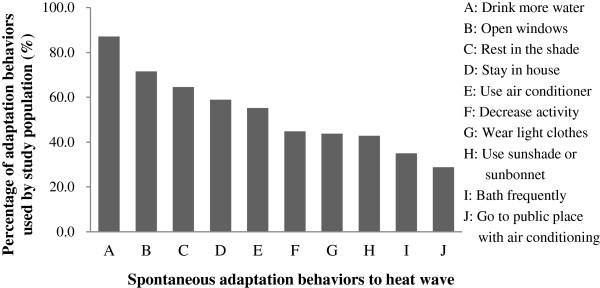
Spontaneous adaptation behaviors to heat waves employed among the public of Guangdong Province.

We also investigated how people usually responded to heat waves. The results showed that 99.1% participants employed at least one spontaneous adaptation behavior during heat waves and that the most common spontaneous adaptation behaviors were “drinking more water”, “opening windows”, and “resting in the shade”. The less commonly reported behaviors were “using a sunshade or sunhat”, “bathing frequently” and “going to a public place with air conditioning” (Figure 2). Older people, those with a lower education level, respondents with a lower family income, those employed in agriculture, forestry, animal husbandry or fishing, and those from rural areas seldom employed behaviors during heat waves such as “opening windows”, “drinking more water”, “wearing light clothes”, “bathing frequently”, “going to a public place with air conditioning”, “using a sunshade or sunbonnet”, “resting in the shade”, and “use an air conditioner”. Furthermore, people with less family income, and from rural areas also rarely used “staying in house” during heat wave days (Table 3). 26.2%, 51.2% and 22.6% respondents usually employed <4, 4–7, and >7 adaptation behaviors during heat waves, respectively (Table 4).

**Table 3:** Spontaneous adaptation behaviors to heat waves in vulnerable populations with different demographic characteristics

	**Open window**		***χ***^**2**^	**P**	**Drink more water**		***χ***^**2**^	**P**	**Wear light clothes**		***χ***^**2**^	**P**	**Bathe frequently**		***χ***^**2**^	**P**	**Stay in house**		***χ***^**2**^	**P**
	**No**	**Yes**			**No**	**Yes**			**No**	**Yes**			**No**	**Yes**			**No**	**Yes**		
	**N (%)**	**N (%)**			**N (%)**	**N (%)**			**N (%)**	**N (%)**			**N (%)**	**N (%)**			**N (%)**	**N (%)**		
**Gender**			1.08	0.298			0.15	0.699			5.13	0.024			0.70	0.404			8.95	0.003
Male	321(27.6)	844(72.4)			148(12.7)	1017(87.3)			681(58.5)	484(51.5)			748(64.2)	417(35.8)			513(44.0)	652(56.0)		
Female	301(29.6)	717(70.4)			135(13.3)	883(86.7)			546(53.6)	472(46.4)			671(65.9)	347(34.1)			384(37.7)	634(62.3)		
**Age (years)**			12.26	0.031			4.60	0.467			17.28	0.004			8.61	0.126			4.84	0.436
<20	64(36.0)	114(64.0)			22(12.4)	156(87.6)			102(57.3)	76(42.7)			108(60.7)	70(39.3)			81(45.5)	97(54.5)		
20–29	135(31.4)	295(68.6)			57(13.3)	373(86.7)			233(54.2)	197(45.8)			291(67.7)	139(32.3)			184(42.8)	246(57.2)		
30–39	138(26.1)	391(73.9)			68(12.9)	461(87.1)			295(55.8)	234(44.2)			355(67.1)	174(32.9)			223(42.2)	306(57.8)		
40–49	145(28.3)	367(71.7)			58(11.3)	454(88.7)			264(51.6)	248(48.4)			316(61.7)	196(38.3)			194(37.9)	318(62.1)		
50–59	73(23.5)	238(76.5)			40(12.9)	271(87.1)			183(58.8)	128(41.2)			195(62.7)	116(37.3)			122(39.2)	189(60.8)		
≥60	67(30.0)	156(70.0)			38(17.0)	185(83.0)			150(67.3)	73(32.7)			154(69.1)	69(30.9)			93(41.7)	130(58.3)		
**Education**			18.25	0.001			16.29	0.003			151.18	<0.001			20.65	<0.001			2.96	0.565
Elementary school or lower	164(31.5)	356(68.5)			82(15.8)	438(84.2)			368(70.8)	152(29.2)			371(71.3)	149(28.7)			215(41.3)	305(58.7)		
Junior middle school	227(32.2)	477(67.8)			99(14.1)	605(85.9)			442(62.8)	262(37.2)			440(62.5)	264(37.5)			275(39.1)	429(60.9)		
Senior middle school or vocational secondary school	134(25.8)	386(74.2)			58(11.2)	462(88.8)			256(49.2)	264(50.8)			309(59.4)	211(40.6)			224(43.1)	296(56.9)		
College or above	81(21.6)	294(78.4)			31(8.3)	344(91.7)			123(32.8)	252(67.2)			258(68.8)	117(31.2)			153(40.8)	222(59.2)		
Didn’t answer	16(25.0)	48(75.0)			13(20.3)	51(79.7)			38(59.4)	26(40.6)			41(64.1)	23(35.9)			30(46.9)	34(53.1)		
**Occupation**			27.77	<0.001			17.76	0.007			138.49	<0.001			36.12	<0.001			6.70	0.350
Agriculture, forestry, animal husbandry or fishing	176(34.7)	331(65.3)			72(14.2)	435(85.8)			367(72.4)	140(27.6)			377(74.4)	130(25.6)			213(42.0)	294(58.0)		
Person in service trade	96(21.4)	352(78.6)			49(10.9)	399(89.1)			214(47.8)	234(52.2)			268(59.8)	180(40.2)			180(40.2)	268(59.8)		
Person in charge of institute	39(25.7)	113(74.3)			14(9.2)	138(90.8)			61(40.1)	91(59.9)			106(69.7)	46(30.3)			68(44.7)	84(55.3)		
Technician	58(23.0)	194(77.0)			20(7.9)	232(92.1)			92(36.5)	160(63.5)			165(65.5)	87(34.5)			93(36.9)	159(63.1)		
Military or student	59(33.1)	119(66.9)			23(12.9)	155(87.1)			98(55.1)	80(44.9)			116(65.2)	62(34.8)			85(47.8)	93(52.2)		
Unemployment or retired	74(29.0)	181(71.0)			36(14.1)	219(85.9)			137(53.7)	118(46.3)			162(63.5)	93(36.5)			100(39.2)	155(60.8)		
Other	120(30.7)	271(69.3)			69(17.6)	322(82.4)			258(66.0)	133(34.0)			225(57.5)	166(42.5)			158(40.4)	233(59.6)		
**Family monthly income per person (yuan)**			63.63	<0.001			17.35	0.008			132.85	<0.001			21.14	0.002			15.51	0.017
< 500	93(32.3)	195(67.7)			40(13.9)	248(86.1)			215(74.7)	73(25.3)			211(73.3)	77(26.7)			118(41.0)	170(59.0)		
500–999	166(43.3)	217(56.7)			67(17.5)	316(82.5)			271(70.8)	112(29.2)			254(66.3)	129(33.7)			146(38.1)	237(61.9)		
1,000–1,999	96(23.3)	316(76.7)			43(10.4)	369(89.6)			200(48.5)	212(51.5)			273(66.3)	139(33.7)			160(38.8)	252(61.2)		
2,000–4,999	112(21.6)	407(78.4)			60(11.6)	459(88.4)			220(42.4)	299(57.6)			326(62.8)	193(37.2)			223(43.0)	296(57.0)		
5,000–9,999	40(23.7)	129(76.3)			20(11.8)	149(88.2)			78(46.2)	91(53.8)			97(57.4)	72(42.6)			62(36.7)	107(63.3)		
≥10,000	16(24.2)	50(75.8)			2(3.0)	64(97.0)			34(51.5)	32(48.5)			33(50.0)	33(50.0)			20(30.3)	46(69.7)		
Don’t know or refused to answer	99(28.6)	247(71.4)			51(14.7)	295(85.3)			209(60.4)	137(39.6)			225(65.0)	121(35.0)			168(48.6)	178(51.4)		
**Residential area**			15.25	<0.001			2.72	0.099			57.74	<0.001			27.70	<0.001			12.86	<0.001
Urban	262(24.6)	802(75.4)			125(11.7)	939(88.3)			510(47.9)	554(52.1)			633(59.5)	431(40.5)			396(37.2)	668(62.8)		
Rural	360(32.2)	759(67.8)			158(14.1)	961(85.9)			717(64.1)	402(35.9)			786(70.2)	333(29.8)			501(44.8)	618(55.2)		
	**Reduce activity**		***χ***^**2**^	**P**	**Go to a public place with air conditioning**		***χ***^**2**^	**P**	**Use sunshade or sunhat**		***χ***^**2**^	**P**	**Rest in the shade**		***χ***^**2**^	**P**	**Use an air conditioner**		***χ***^**2**^	**P**
	**No**	**Yes**			**No**	**Yes**			**No**	**Yes**			**No**	**Yes**			**No**	**Yes**		
	**N (%)**	**N (%)**			**N (%)**	**N (%)**			**N (%)**	**N (%)**			**N (%)**	**N (%)**			**N (%)**	**N (%)**		
**Gender**			0.18	0.671			0.36	0.549			13.85	<0.001			0.73	0.393			0.76	0.385
Male	648(55.6)	517(44.4)			823(70.6)	342(29.4)			710(60.9)	455(39.1)			403(34.6)	762(65.4)			532(45.7)	633(54.3)		
Female	557(54.7)	461(45.3)			731(71.8)	287(28.2)			540(53.0)	478(47.0)			370(36.3)	648(63.7)			446(43.8)	572(56.2)		
**Age (years)**			0.28	0.998			24.29	<0.001			21.17	0.001			4.95	0.422			113.62	<0.001
<20	98(55.1)	80(44.9)			120(67.4)	58(32.6)			100(56.2)	78(43.8)			72(40.4)	106(59.6)			101(56.7)	77(43.3)		
20–29	240(55.8)	190(44.2)			288(67.00)	142(33.0)			230(53.5)	200(46.5)			163(37.9)	267(62.1)			163(37.9)	267(62.1)		
30–39	289(54.6)	240(45.4)			364(68.8)	165(31.2)			287(54.3)	242(45.7)			179(33.8)	350(66.2)			172(32.5)	357(67.5)		
40–49	280(54.7)	232(45.3)			361(70.5)	151(29.5)			284(55.5)	228(44.5)			182(35.5)	330(64.5)			216(42.2)	296(57.8)		
50–59	173(55.6)	138(44.4)			237(76.2)	74(23.8)			196(63.0)	115(37.0)			104(33.4)	207(66.6)			178(57.2)	133(42.8)		
≥60	125(56.1)	98(43.9)			184(82.5)	39(17.5)			153(68.6)	70(31.4)			73(32.7)	150(67.3)			148(66.4)	75(33.6)		
**Education**			4.69	0.321			97.90	<0.001			201.92	<0.001			9.21	0.056			628.21	<0.001
Elementary school or lower	290(55.8)	230(44.2)			443(85.2)	77(14.8)			400(76.9)	120(23.1)			173(33.3)	347(66.7)			415(79.8)	105(20.2)		
Junior middle school	370(52.6)	334(47.4)			521(74.0)	183(26.0)			435(61.8)	269(38.2)			235(33.4)	469(66.6)			395(56.1)	309(43.9)		
Senior middle school or vocational secondary school	290(55.8)	230(44.2)			329(63.3)	191(36.7)			259(49.8)	261(50.2)			208(40.0)	312(60.0)			125(24.0)	395(76.0)		
College or above	214(57.1)	161(42.9)			222(59.2)	153(40.8)			118(31.5)	257(68.5)			129(34.4)	246(65.6)			18(4.8)	357(95.2)		
Didn’t answer	41(64.1)	23(35.9)			39(60.9)	25(39.1)			38(59.4)	26(40.6)			28(43.8)	36(56.3)			25(39.1)	39(60.9)		
**Occupation**			3.11	0.795			104.31	<0.001			177.78	<0.001			34.59	<0.001			544.60	<0.001
Agriculture, forestry, animal husbandry or fishing	283(55.8)	224(44.2)			430(84.8)	77(15.2)			378(74.6)	129(25.4)			151(29.8)	356(70.2)			414(81.7)	93(18.3)		
Person in service trade	234(52.2)	214(47.8)			286(63.8)	162(36.2)			230(51.3)	218(48.7)			142(31.7)	306(68.3)			131(29.2)	317(70.8)		
Person in charge of institute	86(56.6)	66(43.4)			82(53.9)	70(46.1)			64(42.1)	88(57.9)			55(36.2)	97(63.8)			5(3.3)	147(96.7)		
Technician	143(56.7)	109(43.3)			156(61.9)	96(38.1)			79(31.3)	173(68.7)			97(38.5)	155(61.5)			31(12.3)	221(87.7)		
Military or student	95(53.4)	83(46.6)			113(63.5)	65(36.5)			91(51.1)	87(48.9)			67(37.6)	111(62.4)			81(45.5)	97(54.5)		
Unemployment or retired	148(58.0)	107(42.0)			182(71.4)	73(28.6)			138(54.1)	117(45.9)			79(31.0)	176(69.0)			112(43.9)	143(56.1)		
Other	216(55.2)	175(44.8)			305(78.0)	86(22.0)			270(69.1)	121(30.9)			182(46.5)	209(53.5)			204(52.2)	187(47.8)		
**Family monthly income per person (yuan)**			18.62	0.005			54.29	<0.001			121.82	<0.001			35.49	<0.001			596.77	<0.001
< 500	149(51.7)	139(48.3)			234(81.3)	54(18.8)			211(73.3)	77(26.7)			64(22.2)	224(77.8)			254(88.2)	34(11.8)		
500–999	187(48.8)	196(51.2)			305(79.6)	78(20.4)			271(70.8)	112(29.2)			137(35.8)	246(64.2)			254(66.3)	129(33.7)		
1,000–1,999	233(56.6)	179(43.4)			272(66.0)	140(34.0)			214(51.9)	198(48.1)			132(32.0)	280(68.0)			125(30.3)	287(69.7)		
2,000–4,999	306(59.0)	213(41.0)			332(64.0)	187(36.0)			223(43.0)	296(57.0)			202(38.9)	317(61.1)			96(18.5)	423(81.5)		
5,000–9,999	89(52.7)	80(47.3)			120(71.0)	49(29.0)			77(45.6)	92(54.4)			65(38.5)	104(61.5)			26(15.4)	143(84.6)		
≥10,000	30(45.5)	36(54.5)			37(56.1)	29(43.9)			35(53.0)	31(47.0)			26(39.4)	40(60.6)			9(13.6)	57(86.4)		
Don’t know or refused to answer	211(61.0)	135(39.0)			254(73.4)	92(26.6)			219(63.3)	127(36.7)			147(42.5)	199(57.5)			214(61.8)	132(38.2)		
**Residential area**			1.19	0.275			19.27	<0.001			57.04	<0.001			10.53	0.001			404.86	<0.001
Urban	600(56.4)	464(43.6)			711(66.8)	353(33.2)			522(49.1)	542(50.9)			413(38.8)	651(61.2)			243(22.8)	821(77.2)		
Rural	605(54.1)	514(45.9)			843(75.3)	276(24.7)			728(65.1)	391(34.9)			360(32.2)	759(67.8)			735(65.7)	384(34.3)		

**Table 4:** The relationship between health risk perception and spontaneous adaptation behaviors to heat waves in the public of Guangdong Province

	**Number of spontaneous adaptation behaviors to heat waves**			**χ**^**2**^	**P**
	**<4**	**4–7**	**>7**		
	**N(%)**	**N(%)**	**N(%)**		
Health risk perception to heat waves				166.52	<0.001
Low impact	161(49.7)	118(36.4)	45(13.9)		
Moderate impact	365(25.6)	761(53.4)	299(21.0)		
High impact	45(10.4)	239(55.1)	150(34.6)		

The health risk perception level was positively related to the number of spontaneous adaptation behaviors (χ^2^ =166.52, *p*<0.001), indicating that the higher the risk perception of the heat event impact, the more spontaneous adaptation behaviors individuals employed (Table 4).

### Associations between risk perception, spontaneous adaptation behaviors and heatstroke in the past year

A total of 149 (6.8%) respondents reported that they had experienced heatstroke in the past year. Unconditional logistic regression analyses showed that, after adjustment for potential confounders (including age, gender, education, occupation, family income and residential area), participants who perceived heat waves to be a moderate (OR=2.93, 95% CI: 1.38-6.22) or high risk (OR=10.58, 95% CI: 4.74-23.63) to their health experienced more heatstroke in the past year compared with those with a low health risk perception. Of all of the participants, people who wore light clothes during heat waves experienced less heatstroke (OR=0.42, 95% CI: 0.27-0.67), but people who open windows (OR=2.21, 95% CI: 1.38-3.54) or rested in the shade (OR=1.65, 95% CI: 1.09-2.51) reported more heatstroke. Stratified analyses revealed that among participants from urban areas heatstroke was negatively associated with drinking more water (OR=0.37, 95%CI: 0.14-0.94), and wearing light clothes (OR=0.39, 95%CI: 0.16-0.96), but positively associated with bathing frequently (OR=3.11, 95%CI: 1.32-7.33). Among individuals from rural areas heatstroke was negatively related to decreasing activity (OR=0.55, 95% CI: 0.33-0.92), and wearing light clothes (OR=0.41, 95% CI: 0.24-0.70), but positively related to opening windows (OR=2.87, 95% CI: 1.61-5.08) (Table 5).

**Table 5:** Associations of heatstroke with each spontaneous adaptation behavior in the public of Guangdong Province

	**Total**	**Urban areas**	**Rural areas**
	**Adjusted OR**^**† **^**(95% CI)**	**Adjusted OR**^**† **^**(95% CI)**	**Adjusted OR**^**† **^**(95% CI)**
Drink more water			
No	1	1	1
Yes	0.95 (0.56–1.63)	0.37* (0.14–0.94)	1.39 (0.71–2.72)
Open windows			
No	1	1	1
Yes	2.21* (1.38–3.54)	0.94 (0.38–2.35)	2.87*(1.61–5.08)
Rest in the shade			
No	1	1	1
Yes	1.65* (1.09–2.51)	1.00 (0.45–2.23)	1.63 (0.96–2.77)
Stay in house			
No	1	1	1
Yes	0.70 (0.47–1.04)	0.71 (0.31–1.60)	0.79 (0.49–1.26)
Use air conditioner			
No	1	1	1
Yes	0.71 (0.46–1.09)	0.89 (0.36–2.21)	0.97 (0.59–1.60)
Decrease activity			
No	1	1	1
Yes	0.68 (0.45–1.04)	0.98 (0.43–2.25)	0.55* (0.33–0.92)
Wear light clothes			
No	1	1	1
Yes	0.42* (0.27–0.67)	0.39 (0.16–0.96)	0.41 (0.24–0.70)
Use sunshade or sunhat			
No	1	1	1
Yes	0.85 (0.55–1.32)	2.11 (0.90–4.94)	0.78 (0.45–1.36)
Bath frequently			
No	1	1	1
Yes	1.42 (0.93–2.15)	3.11* (1.32–7.33)	1.21 (0.72–2.03)
Go to public place with air conditioning			
No	1	1	1
Yes	0.99 (0.61–1.60)	1.04 (0.43–2.52)	0.90 (0.49–1.65)

† Logistic regression adjustment for age, gender, education, occupation, family income and residential area.

*: P<0.05.

To test the interactive effects between risk perception and adaptation behaviors on heatstroke, we divided all participants into three groups according to the number of adaptation behaviors during heat waves. The main effect analyses showed that people who employed 4–7 (OR=0.38, 95% CI: 0.25-0.57) or >7 (OR=0.47, 95% CI: 0.28-0.78) spontaneous adaptation behaviors during heat waves had a lower risk of heatstroke than those who used <4 adaptation behaviors to heat waves. Stratified analyses further found that health risk perception and the number of spontaneous adaptation behaviors to heat waves had negative interactive effects on heatstroke experiences (*P*=0.014 and 0.001 for interactive effect tests of high impact × 4–7 adaptation behaviors and high impact × >7 adaptation behaviors, respectively), and people with high risk perception and employing <4 spontaneous adaptation behaviors during heat waves had the highest risk of heatstroke in the past one year (OR=47.46, 95% CI: 12.82-175.73) of all eight groups. More than half people in that group reported heatstroke in the last year (Table 6).

**Table 6:** Main and interactive effects between health risk perception and spontaneous adaptation behaviors to heat waves on heatstroke experience in the public of Guangdong Province

	**Heat stroke experience in the past one year**				
	**Controls N (%)**	**Cases N (%)**	**Model I**	**Model II**	**Model III**
			**Unadjusted OR (95% CI)**	**Adjusted OR**^**† **^**(95% CI)**	**Adjusted OR**^**† **^**(95% CI)**
Health risk perception of heat waves					
Low impact	315(97.5)	8(2.5)	1	1	
Moderate impact	1336(93.8)	89(6.2)	2.63(1.26–5.46)	2.93(1.38–6.22)	
High impact	382(88.0)	52(12.0)	5.36(2.51–11.45)	10.58(4.74–23.63)	
Number of spontaneous adaptation behaviors to heat waves					
<4	510(89.5)	60(10.5)	1	1	
4–7	1060(94.8)	58(5.2)	0.47 (0.32–0.68)	0.38 (0.25–0.57)	
>7	463(93.7)	31(6.3)	0.57 (0.36–0.89)	0.47 (0.28–0.78)	
Health risk perception × numbers of spontaneous adaptation behaviors to heat waves					
Low impact×<4 adaptation behaviors	157(98.1)	3(1.9)			1
Low impact×4–7 adaptation behaviors	115(97.5)	3(2.5)			1.39(0.27–7.10)
Low impact×>7 adaptation behaviors	43(95.6)	2(4.4)			3.40(0.54–21.43)
Moderate impact×<4 adaptation behaviors	331(90.7)	34(9.3)			4.99(1.50–16.63)
Moderate impact×4–7 adaptation behaviors	725(95.3)	36(4.7)			2.25(0.68–7.47)
Moderate impact×>7 adaptation behaviors	280(93.6)	19(6.4)			3.58(1.03–12.44)
High impact×<4 adaptation behaviors	22(48.9)	23(51.1)			47.46(12.82–175.73)
High impact×4–7 adaptation behaviors	220(92.1)	19(7.9)			6.86(1.95–24.80)
High impact×>7 adaptation behaviors	140(93.3)	10(6.7)			5.46(1.43–20.80)
P for interaction of high impact×4–7 adaptation behaviors					0.014
P for interaction of high impact×>7 adaptation behaviors					0.001

† Logistic regression adjustment for age, gender, education, occupation, family income and residential area.

## Discussion

Although heat waves or extremely high temperatures have been recognized as one major health risk by many previous studies on relationship between temperature and health [20-23], few studies have examined the public’s risk perception of heat waves. Public risk perceptions of heat waves can fundamentally compel or constrain political, economic and social actions to address the risks and dangers from global or regional heat wave events [24]. In addition, Simon reviewed heat events by region and country during the period 2000–2007, finding that China was one of the most severely impacted regions [25]. It is crucial to understand the public’s risk perception of heat waves in China because risk perception might be an important predictor of adaptation and behavior change in previous studies [26]. In the present study, we found that a majority of people (85.2%) in Guangdong province thought heat waves were a moderate or large threat to their health. This finding was comparable to the results in Kalkstein et al. study conducted in Phoenix, Arizona, USA [11]. They assessed the public’s perceived risk of heat waves and found that over 90% of respondents reported heat was dangerous or very dangerous to them [11]. We also observed a lower risk perception of heat waves in males, young people and the elderly, people with less education, those with either extremely low or extremely high family income, those employed in agriculture, forestry, animal husbandry or fishing, and those living in rural areas. This observation was similar to some findings from previous studies [26-28]. Although less knowledge was thought to be a reason for the lower risk perception of extreme weather events [29], some other reasons might also partially explain these findings. For example, males, rural populations, and employees working outdoors usually thought that they were strong enough to withstand heat waves and thus did not take appropriate adaptation measures if exposed to heat waves [19]. Therefore, further studies are needed on how to effectively improve the risk perception level in different populations. These research could provide information to improve the adaptive capacity within a population.

Adaptation is another important component in vulnerability assessment. Improving adaptation capacity could reduce vulnerability to adverse impacts of climate change, and hence minimize the related burdens [30]. In the present study, we found that, although the vast majority of respondents used at least one spontaneous adaptation behavior during heat waves, some important adaptation behaviors such as “going to a public place with air conditioning”, “using a sunshade or sunhat”, and “using an air condition” were less commonly used. Furthermore, some vulnerable groups used fewer adaptation behaviors during heat waves. These vulnerable groups included the elderly, people with a lower education, less income, employed in agriculture, forestry, animal husbandry or fishing, and from rural areas, which may partially explain the higher prevalence of heatstroke experienced in these groups. Some reasons may account for these results. Firstly, some residents had a low risk perception, hence they were simply unaware that they were at risk and didn’t change their behaviors [31]. As described in the Model of Private Proactive Adaptation to Climate Change (MPPACC) developed by Grothmann et al., risk perception is an important psychological dimension of adaptation, and a higher risk perception may predict better adaptation [30]. Our study also showed a positive relationship between risk perception and the number of adaptation behaviors. Secondly, some populations may have less access to some adaptation behaviors. For example, people on extremely low incomes rarely buy air conditioners because of the high cost, and usually employ evaporative coolers during heat waves, which are less effective in protecting people from hot weather [11]. In addition, even if some families had air conditioners, the high cost of electricity often prohibited them from using them often. Thirdly, some adaptation behaviors such as using air conditioners are not practical for some populations such as farmers working on the land, and construction workers working on building sites. Fourthly, the elderly did not usually change their behaviors during heat waves, because most thought they did not belong to the vulnerable category [19]. This kind of unrealistic cognition is usually called optimistic bias which may lead to avoidant maladaptation, and cause themselves more vulnerable to heat waves [32,33]. These results indicated to us that targeted strategies and measures are still needed to promote adaptation capacity in these vulnerable groups. Possible strategies include increasing communication about heat waves to this group and assisting individuals to respond to heat waves by developing cooling centers and opening them free of charge to the elderly during heat wave days, increasing subsidies for buying air conditioners, and decreasing electrovalence on air conditioning.

To test the associations of risk perception and the number of spontaneous adaptation behaviors with heatstroke experience, we used a series of multivariate logistic regressions to assess their main and interactive effects on heatstroke. We found that people with a higher risk perception to heat waves had more heatstroke, which was consistent with the Vitek et al. theory model [34]. This model suggests that people’s risk perception of a disaster is influenced by their own experience of disasters [34]. Such personal experience could reduce their apathy, indifference, wishful thinking and denial, all of which can make communication about risky events more meaningful and important to them, and hence lead to an increase in their risk perception [9,35]. However, due to the limitations of our cross-sectional survey, we can’t confirm whether high risk perception or heatstroke experience came first. Therefore, prospective studies are needed to further test the relationships between risk perception and heatstroke.

In addition, we observed that spontaneous adaptation behaviors associated with lower heatstroke varied between populations from urban and rural areas. This is important for mounting intervention and prevention strategies against the health impacts of heat waves in different contexts. For example, people could be encouraged to drink more water during heat waves to protect themselves from heatstroke. However, in rural areas drinking more water may be not enough to protect people, and other more effective measures will be needed, such as decreasing activity and wearing light clothes. Moreover, we also found that some adaptation behaviors were positively related to heatstroke, which was reverse to what we expected. This difference may be partially explained by our research design, because the present cross-sectional design protected us from clarifying the causal relationship between adaptation behaviors and heatstroke. On the other hand, these results also provided some significant information for heatstroke intervention and prevention. For instance, the positive relationship between opening windows and heatstroke indicated that opening windows during heat waves might increase people exposure to high temperatures, and hence increase their risk of heatstroke. Therefore, closing windows and using some other adaptation behaviors, such as air conditioners, might protect people more effectively.

Another important finding showed that the number of employed spontaneous adaptation behaviors was negatively related to heatstroke risk indicating that the greater the number of adaptation behaviors used during the heat wave days the lower prevalence of heatstroke among the public. Therefore, risk communication strategies should focus on promoting a range of multiple adaptation behaviors to deal with heat waves.

Finally, we found that risk perception and the number of spontaneous adaptation behaviors to heat waves had negatively interactive effects on heatstroke experiences, and people with high risk perception and employing fewer than 4 spontaneous adaptation behaviors during heat waves (n=45) had the highest risk of heatstroke in the past one year. This result indicated that although most people in this subgroup had experienced heatstroke, they also knew that a heat wave was a serious threat to their health but seldom employed enough adaptation behaviors to deal with heat waves. To look for some possible reasons to explain this result, we further analyzed the demographic characteristics of this group of people (Table 7). It was revealed that most of them came from rural areas, had a lower income and less education, and were employed in agriculture, forestry, animal husbandry or fishing. They probably did not have enough income to buy air conditioners, had to work to make a living during heat wave days, or did not know how to adapt to heat waves. Therefore, more attention should be directed to these vulnerable populations, and some special adaptation strategies are needed for these groups.

**Table 7:** Demographic characteristics of the people with high health risk perception but employing <4 spontaneous adaptation behaviors during heat waves (n=45)

	**N**	**(%)**
**Gender**		
Male	27	60.0
Female	18	40.0
**Age (years)**		
<30	10	22.3
30–49	20	44.4
≥50	15	33.3
**Education**		
Elementary school or lower	22	48.9
Higher education	23	51.1
**Occupation**		
Agriculture, forestry, animal husbandry or fishing	22	48.9
Other occupations	23	51.1
**Family monthly income per person (yuan)**		
< 500	27	60.0
≥ 500	18	40.0
**Residential area**		
Urban	14	31.1
Rural	31	68.9

### Study limitations

This study is a cross-sectional study, which limited our ability to infer a causal relationship and identify whether risk perception influenced heatstroke incidence or vice versa. Secondly, all heatstroke cases were self-reported, but not always specifically diagnosed by a medical professional. This may have led to an inaccurate estimation of heatstroke reporting. Thirdly, risk perception was assessed by one question but not a standard scale. Participants probably had different understandings of this question, which may have led to information bias. However, all the interviewers were well trained, and tried to interpret the definition of heat wave consistently for participants, which could increase the validity and reliability of collected information. Fourthly, all the adaptation behaviors were treated with equal weight, which might lead to information bias. Furthermore, exploring the best combination of adaptation behaviors could provide more information for intervention and prevention of heatstroke. However, all the adaptation behaviors mentioned in the present study were the commonly used during heat waves. We don’t know which combination of adaptation behaviors is practical and reasonable in people’s everyday life, which prevented us from investigating the effective combination of adaptation behaviors against heatstroke during heat waves. Therefore, further studies will be needed to focus on this topic in the future. Finally, this investigation was conducted from September to November 2010 and the hottest period is usually in July in Guangdong province. There may have led to recall bias due to the time difference between the heat wave periods and the data collection period.

## Conclusions

There is a large room for improving the health risk perception and adaptation capacity to heat waves among the public of Guangdong province. People with a higher risk perception and who employed fewer adaptation behaviors during heat waves may be more vulnerable to heat waves, and need more attention to improve their adaptation capacity. Our key recommendations are that: (a) risk communication strategies and adaptation planning need to be further developed to improve the risk perception and adaptation capacity of climate change in the public of Guangdong province. (b) assessments of the public’s health vulnerability assessment to heat waves should be carried out to improve the adaptation capacity of the most vulnerable populations.

## Abbreviations

MPPACC: Model of private proactive adaptation to climate change; OR: Odds ratio; CI: Confidence interval; USA: United States of America; SD: Standard; IPCC: Deviation; intergovernmental panel on climate change.

## Competing interests

The authors declared that they have no competing interests.

## Authors’ contributions

TL participated in the design of this study, analyzed the data and drafted the manuscript. YJX participated in the design of the study and data collection. YHZ participated in the design of the study. QHY participated in the data collection. XLS participated in the data collection. HYX participated in the data collection and data analysis. YL participated in the data collection and data analysis. SR participated in the design of the study and draft revision. CC participated in the design of the study and draft revision. HLL participated in the design of the study and data analysis. WJM conceived of the study, and participated in its design and coordination. All authors read and approved the final manuscript.

## Pre-publication history

The pre-publication history for this paper can be accessed here:

http://www.biomedcentral.com/1471-2458/13/913/prepub

## Supplementary Material

Additional file 1 Investigation on the risk perception and adaptation behaviors to heat waves among the public of Guangdong Province. Click here for file
