# Supplementary material for: Associations between risk perception, spontaneous adaptation behavior to heat waves and heatstroke in Guangdong province, China
Source: BMC Public Health. 2013 Oct 2;13:913. doi: 10.1186/1471-2458-13-913 (PMC3853971; doi:10.1186/1471-2458-13-913)
Supplement: Additional file 1 — Investigation on the risk perception and adaptation behaviors to heat waves among the public of Guangdong Province. [file 1471-2458-13-913-S1.doc]

**Investigation on the risk perception and adaptation behaviors to heat waves among the public of Guangdong province (*Appendix*)**

**Part Ⅰ. Family questionnaire**

A01. Family code：□□□□□

A02. Administrative division code：□□Province □□City □□County/District

A03. Town/street code: □□

A04. Village/community code：□□

A05. Sub-area code：□□□

A06. Family address：

A07. Name of the householder：

A08. Connect number：

A09. How many members in your family (including all people who take your house as their main living places recently)?

□□ Persons

A10. How many members aged 15-69 years in your family?

□□ Persons

A11. General information of all people aged 15-69 years in your family.

| a. Name | b. Age  (Years) | c. Gender  □1 Male □2 Female | d. Individual code | e. Use ‘√’to mark the one who will participant the individual interview |
| --- | --- | --- | --- | --- |
|  | □□ | □1 □2 | □□□□□□ |  |
|  | □□ | □1 □2 | □□□□□□ |  |
|  | □□ | □1 □2 | □□□□□□ |  |
|  | □□ | □1 □2 | □□□□□□ |  |
|  | □□ | □1 □2 | □□□□□□ |  |
|  | □□ | □1 □2 | □□□□□□ |  |
|  | □□ | □1 □2 | □□□□□□ |  |
|  | □□ | □1 □2 | □□□□□□ |  |
|  | □□ | □1 □2 | □□□□□□ |  |
|  | □□ | □1 □2 | □□□□□□ |  |

Name of the investigator： Signature:

Date: Year Month Day

**Part Ⅱ. Personal questionnaire**

**Section 1. Socio-demographic characteristics**

B01. Individual code: □□□□□□

B02.Your sex: □1. Male □2. Female

B03. When is your birthday?

Which year：□□□□ （**If do not know, please fill in “9999”**）

Which month：□□ （**If do not know, please fill in “99”**）

（***Note: If the individual does not remember their birthday exactly, please continue to finish question A04, or jump to question A05***）

B04. How old are you?（***Note: if the individual does not remember their own age, please ask their family members***）

□□□ Years

B05. What is your final education level?

□1. Elementary school or lower □2. Junior middle school

□3. Senior middle school or vocational secondary school

□4. College or above □5. Refuse to answer

B06. What is your main occupation during the past 12 months?

□1. Agriculture, forestry, animal husbandry or fishing □2. Person in service trade

□3. Person in charge of institute □4. Technician

□5. Military and student □6. Unemployment and retirement

□7. Others__________________________

B07. What was the average per capita income in your family (yuan, after tax) during the past 12 months?

□1. < 500 □2. 500-999 □3. 100 0-1999 □4. 2000-4999

□5. 5,000-9,999 □6. ≥10,000 □7 Don’t know or refused to answer

**Section 2. Risk perception and adaptation behaviors to heat waves**

C01. Have you heard about heat waves in the past (***a “heat day” is defined as a day with maximum temperature exceeding 35℃, and a heat wave is a period with at least three consecutive “heat days”***)?

□1 Yes

□2 No

C02. In the past several years, did you feel the weather was hotter than before?

C021. What are the reasons for these changes in your opinion ([***Multiple***](app:ds:multiple)***-***[***choice***](app:ds:choice) ***question***)?

□1. Humanity’s CO_2_ emissions

□2. Air pollution

□3. Ozone hole in the atmosphere

□4. Natural law

□5. Others

□6. Do not know

Continue

□1. Yes

□2. No

□3. Not sure

C03. Have you heard about heat waves warnings in the past（yellow, orange or red warning）？

Continue

C031. What are the main channels through which you heard the heat wave warning information？

□1. Newspaper □2. Television □3. Radio

□4. Internet □5. Friends □6 Others

C032. Did you usually care about this heat wave warning information?

□1. Very much □2. A little □3. General level

□4. Very little □5. Not at all

□1. Yes

□2. No

C04. How dangerous are heat waves to your health？

□1. Absolutely not

□2. Very small

□3. Small

□4. Generally

□5. Strong

□6. Very strong

□7. Extremely

C05. What are your commonly used spontaneous adaptation behaviors during the heat waves ([***multiple***](app:ds:multiple)***-***[***choice***](app:ds:choice) ***question***)?

□1.Open windows

□2. Drink more water

□3. Wear light clothes

□4. Bath frequently

□5. Stay in house

□6. Decrease activity

□7. Go to public place with air conditioning,

□8. Rest in the shade,

□9. Use air conditioner,

□10. Use sunshade or sunhat

**Continue**

□11. None

C051 What is the main reasons for not changing your behaviors during the heat waves?

□1 The weather was not hot enough

□2 Heat waves had no effect on me

□3 I am used to the hot weather

□4 I have no capacity to change my behaviors

□5 Other

C06. Have you been diagnosed with heatstroke by a doctor or had any symptoms of heatstroke during the heat waves periods in the past one year（***including dizziness, headache,*** [***nausea***](app:ds:nausea)***,*** [***vomiting***](app:ds:vomiting)***, chest stuffiness, palpitation, and muscle spasm. All the symptoms due to other reasons should be excluded, such as intestinal infectious disease***）？

C061. Have you been treated in hospital for heatstroke in the past one year?

□1. Yes

□2. No

□3. Don’t remember

Continue

□1 Yes

□2. No

□3. Not sure

Name of the investigator： Signature:

Date: Year Month Day
